# A Method to Redesign and Simplify Schedules of Assessment and Quantify the Impacts. Applications to Merck Protocols

**DOI:** 10.1007/s43441-024-00666-x

**Published:** 2024-05-10

**Authors:** Steven R. Cummings, Scott Chetham, Andy Lee

**Affiliations:** 1 San Francisco Coordinating Center, California Pacific Medical Center Research Institute, Mission Hall: 550-16th Street, 2nd Floor, 94143 San Francisco, CA USA; 2 Department of Epidemiology and Biostatistics, University of California, Mission Hall: Box #0560 550- 16th Street, 2nd Floor, 94143 San Francisco, CA USA; 3 Faro Health Inc, 3366 N Torrey Pines CT, Suite 225, 92037 La Jolla, CA USA; 4 Global Clinical Trial Operations, Merck & Co., Inc, Rahway, NJ USA

**Keywords:** Clinical trial, Research design, Complexity, Cost, Simplification, Participant burden

## Abstract

The growing complexity of biopharmaceutical sponsored trials has adverse impacts on increased burdens on participants, clinical sites, and sponsors, including greater difficulty recruiting and retaining participants, difficulty engaging sites to participate in trials, excessive cost of trials, and increased cycle times. The schedule of assessments (SoAs) is the origin of and blueprint for complexity that is often generated by copying and pasting from previous SoAs. We developed an approach, termed Lean Design, for redesigning the assessments in SoAs that generate data, the ‘Data SoA.’ It starts with a simple “ground zero” SoA. Any addition is challenged using several principles of trial design. We employed a system, the Faro Trial Designer Tool, to quantify the impacts of changes in an SoA to provide real-time feedback to the team and sponsor. We applied the approach in workshops with teams for six clinical trials in various stages of design and implementation. The approach resulted in recommendation for substantial potential savings in participant and site staff time, costs, and complexity of the trials. Application of this approach to very early stages of protocol design has the potential to reduce the complexity of biopharmaceutical sponsored trials and its consequences.

## Introduction

Industry-sponsored clinical trials have become excessively complex. The growing amount of data to collect, monitor, clean, process, analyze, interpret, and review by regulators has also contributed to increases in the mean duration of both Phase II and Phase III clinical trials [1]. The burden of trials to participants is increasing the difficulty of recruiting and retaining participants. The burden is greatest for people in lower socioeconomic and minority groups with inflexible job schedules and lack of childcare. Burden on staff diminishes the attractiveness of trials to clinical sites.

ICH guideline E19 encourages sponsors to reduce the amount of data collected and submitted for review that has little or no relevance to indications to treat and plausible adverse effects from the investigational product (IP) [1]. However, little progress has been made in reducing the complexity of protocol design and new approaches are fundamentally required [2]. 

Clinical development teams receive remarkably little feedback concerning the impacts of their choices on the time required of study participants, the complexity of the trial for staff, and the cost of elements included in the trial. Those metrics would also be valuable to the Sponsor of the trial to monitor the cost and impacts of protocols and to consider ways to reduce them. Faro Health Inc. (www.farohealth.com) has developed a tool that quantifies key aspects of a trial that reveal the impact of choices made in the design of trial protocols.

A major source of increasing complexity is reliance on the process of “Copy-and-Paste.” The most efficient approach to creating a schedule of assessment (SoA) is to copy one from previous trials or a standard template. The designer typically adds additional assessments for a variety of reasons, including secondary and exploratory endpoints, measurements about the mechanism of the drug effect, or assessments of quality of life. Some assessments may be added in anticipation to questions from regulatory agencies.

Reviews of protocols by committees typically focus, for example, on how the trial will increase the likelihood of successful registration, feedback from thought leaders, cost of the trial, or sufficient assessment of safety. Reducing the complexity of the SoAs is not commonly a consistent focus of internal reviews. The SoAs that emerge from the process become templates for other trials. Thus, complexity snowballs without constraint.

Studies of the costs of clinical trials have shown that predictable factors, including the number of participants, sites, and the duration of trials all influence the cost of trials [3–6]. No study to date has measured the contributions of inefficiencies of protocol design to the cost of trials. Further, no studies have been conducted to investigate the impact of inefficient trial design on the hours required of patients to participate in a trial and clinical staff to conduct them. Most importantly, no study has demonstrated how much reduction in unnecessary features of the SoA may reduce burdens and costs. Merck has undertaken a project, termed ‘Lean Design’ to simplify trials and quantify the potential impacts.

## Methods

### Lean Design

The Lean Design exercise uses only a schedule of assessment (SoA), a spreadsheet that includes all the assessments for data collection that would be done in the trial. The Lean Design process focuses on assessments in the SoA that generate data. In that sense, it may be referred to as the Data SoA. It does not include treatments or administrative procedures, such as accounting for study drug. Furthermore, the Lean Design exercises do not include recruitment and screening procedures. These warrant different considerations.

**Fig. 1:**
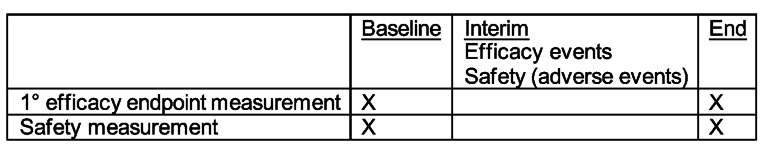
Basic “Ground Zero” schedule of assessments for data collection

The Lean Design process starts with a basic minimum set of assessments: “Ground Zero” (Fig. 1) The primary endpoint that defines the sample size of the trial is not changed. Reporting of adverse events is also retained. Additions to a basic SoA are considered and may be challenged. We assume that this approach of starting with a very simple SoA will result in substantially simpler SoAs than would the common approach of considering items that are already part of an established complex SoA. This concept is based on the principle of “Anchoring Bias” (https://www.verywellmind.com/what-is-the-anchoring-bias-2795029), that the starting point for decisions strongly influences the outcome of deliberations and negotiations (Table 1).

**Table 1:** Selected lean design process and principles for developing schedules of assessments (SoA) for data collection

1. Specify the primary endpoint and sample size
2. Start with an otherwise blank SoA (no data generating elements, e.g., lab tests)
3. Propose assessments one-by-one for the new SoA.
4. Challenge potential additions.
- Should it be included? Is there a biological basis for an effect of the treatment on the proposed assessment? If not, the item is not included in the SoA.
- If included, when should the assessments be done?
o If the effects have an early onset, assess at three points: baseline, peak effect, and end of follow-up
o If the effects are cumulative, consider assessing twice, at baseline and end of follow-up.
- In how many participants should this assessment be made? (The sample size for an assessment, such as for a secondary endpoint, will generally differ from that required for the primary endpoint)
o If fewer than the total, make the measurement in a subset of participants.
5. Safety assessments: Differentiate two types: individual participant safety or characterizing the treatment
- Individual participant safety. A value of the assessment would change treatment or require an intervention.
- To characterize the off-target effects of the molecule
o Perform in a sample of participants large enough to detect an important change.
o Perform once or twice after baseline depending on time course of biological effects: early for peak effects or at end of trial for cumulative effects.
6. Assessments for clinical care may not generate data
o Physical examinations, including routine vital signs at specified frequencies, may be included in clinical care. Do not include them in the Data SoA.
o Some examinations may collect specific data, such as whether cancer has recurred
o Periodic clinical care, included in the protocol, may capture important unanticipated findings as treatment emergent adverse events.
7. Select only relevant items from panels of tests
- Enter only the intended measurement in the EDC.

### Application to Specific Protocols

Merck clinical trial leadership selected six protocols from different therapeutic areas for Lean Design workshops: cardiovascular disease and other medical conditions, psychiatry, and oncology. Initial workshops oriented the team to the Lean Design method. These were followed by in-person workshops with the study team that reviewed the SoA using the Lean Design principles. Workshops were led by an expert in clinical trial design and a member of the Faro team.

Teams were told that the workshops were exercises and changes recommended in workshops were not mandatory. Most protocols were in late stages of development and approval which precluded making many of the changes that were recommended or agreed to in the workshops.

To the extent possible, workshop leaders created a very simple Ground Zero version of the data-generating sections of the SoAs (“Data SoA”). They removed laboratory tests, physical examinations, clinical assessments, patient reported outcomes (PROs) and other assessments.

Measurements for the primary endpoint and collection of adverse events were retained. The elements of the SoA that did not generate data, were not included.

The study teams then proposed additions to the SoA. Potential additions were then challenged as to whether there were plausible biological effects of the investigational drug on an assessment. A proposal to include a complete blood count was considered based on plausible effects of the treatment on any components of the blood count. Second, if there was a biological basis for an effect, then the timing and frequency of the measurements was considered. If the drug had a rapid onset of effect(s) that remained constant, then a measurement at the time of anticipated peak effect was included and the rationale for subsequent measurements was questioned. If the drug was expected to have cumulative effect(s), for example, effect on the progression of fibrotic changes on imaging, it was recommended that the assessment would be done at baseline and once at the end of treatment or of the observation period.

The exercise did not question the total sample size for the trial. However, the sample size for a trial is based on the expected effect size for the primary endpoint, Thus, that sample size may not be relevant to other assessments. For example, some assessments, such as a laboratory test value, needed a smaller sample size to find important effects. In these instances, the assessment could be done in subsamples of participants. For large trials, it was pointed out that some assessments that were proposed for addition to the SoA for everyone could be done in smaller subsets while preserving sufficient statistical power to find important effects. Strategies that were proposed included making assessments in the first participants or in a sample of clinical sites.

Two types of safety assessments were identified. First, assessments for individual participant safety that would need to be done periodically in all participants to discover actionable abnormalities. For example, for a drug with treatment of psychiatric effects that might increase the risk of suicide, then the development of a suicidal ideation would be made in all participants frequently enough to detect suicidal ideations before a suicide attempt. Second, all other assessments would be done to characterize the profile of adverse effects. It was noted that these could be done in limited sample sizes sufficient to detect important effects and performed only at times consistent with the time course of the biological effects of the treatment.

The workshop leaders challenged the inclusion of assessments for routine clinical care, such as physical exams. Generally, the physical examinations did not specify data to be collected. Thus, they may be part of the protocol but, rather than including them as assessments in the Data SoA, any abnormalities discovered by clinical examinations can be reported as adverse events. Some physical examinations collect essential and specified data, such as examination of sites of cancer recurrence to assess progression-free survival. In these instances, the specific assessments included in the Data SoA.

Frequently, panels of tests, such as chemistry panels are proposed for the purpose of making one or two measurements, such as creatinine for renal function. However, they include many other measurements, such as magnesium or uric acid, that are not relevant to the treatment or condition.

The workshop leaders pushed back against several common reasons for including activities that were not allowed in the construction of new SoAs. These reasons included the following: the activity is a standard part of protocols, the participant is already at the clinical site, the assessment is part of routine medical care for the condition, or a sample has already been obtained (for a different purpose). The same principles were applied to laboratory and physical measurements and questionnaires or interviews, for example, for patient-reported outcomes (PROs). Most PROs are collected in all trial participants without considering intervention effect or sample size. If the total sample size for the trial may not be large enough to find important effects on the metric of quality of life, then the likelihood of an underpowered negative result may mean of inclusion of the instrument is not worth the time required of participants and staff. The workshop leaders also challenged the inclusion of PK/PD sampling in all participants at multiple visits without a sample size rationale.

Study teams had “homework” follow-up from the workshop. Study teams met to discuss recommendations from the workshop, and which changes they would adopt. In a follow-up meeting, the teams reported their revised version of the SoA to the leaders of the Lean Design workshop. Reasons for retaining assessments that had not been recommended in the Workshop were discussed.

### Quantifying the Impact of SoAs: The Faro Smart Design Tool

The Faro Smart Design Tool (Faro Health Inc.) generated estimates of the the patient burden, patient visit times, required site staff time, activity cost, blood volumes and operational complexity for a site. The Faro team quantified the changes in the impacts of the SoA, comparing the original to a version that represented the recommendations and proposed changes made during the workshop. Using the tool and extensive databases of hours, direct activity costs, and complexity of the three versions and differences between the original, the ‘Workshop,’ and the version with changes that the team adopted.

The Faro tool draws data from many real world sources. Data accuracy in the Faro tool is ensured throughout the data lifecycle by programmed validations, as well as detailed review of the real world data sources to confirm this data is correctly represented. At each release of new functionality, the cloud based software is validated to confirm the quality of existing and new functionality.

## Results

The Lean Design workshops usually generated major potential changes in SoAs that would result in substantial savings in participant and staff time, costs, and complexity calculated from the Smart Design Tool (Tables 2, 3 and 4). Accumulated across numerous trials in a sponsor’s portfolio, durable effects of the Lean Design method would have substantial effects on the burdens and costs and cycle times of a sponsor’s clinical trials.

**Table 2:** Impact of lean design workshops on patient hours

Therapeutic Areas Number of Participants. Duration of Trials (wks.)	Baseline and Change in Patient Hours (hrs.)					
	Per Participant			Total for Trial		
	Pre Workshop	Result of Workshop	Adopted	Pre Workshop	Result of Workshop	Adopted
Liver Disease *N* = 328, 78 wks.	40.4	-15.10	-3.7	13,251	-4,953	-1,213
Cardiac Disease *N* = 12,600, 250 wks.	47.8	-9.4	-4.3	602,280	-118,440	-54,180
Pulmonary Disease *N* = 164, 122 wks.	395.2	-47.2	-9.3	64,813	-7,734	-1,525
Cancer *N* = 1170, 128 wks.	168.5	-6.0	0.0	197,145	-7,020	0
Infectious Disease *N* = 2596, 24 wks.	24.3	-4.0	-4.8	63,083	-16,932	-12,461
Psychiatric Disease *N* = 500, 15 wks.	45.4	-7.9	-5.8	22,700	-4,040	-2,900

**Table 3:** Impact of lean design workshops on hours spent by site staff

Therapeutic Areas Number of Participants. Duration of Trials (wks.)	Baseline and Change in Site Staff (hrs.)					
	Per Participant			Total for Trial		
	Pre Workshop	Result of Workshop	Adopted	Pre Workshop	Result of Workshop	Adopted
Liver Disease *N* = 328, 78 wks.	60.70	-29.10	-3.70	19,910	-9,545	-1,214
Cardiac Disease *N* = 12,600, 250 wks.	68.30	-9.10	-4.80	860,580	-114,660	-60,480
Pulmonary Disease *N* = 164, 122 wks.	108.80	-46.30	-2.10	17,843	-7,588	-344
Cancer *N* = 1170, 128 wks.	195.90	-6.00	0.00	229,203	-7,020	0
Infectious Disease *N* = 2596, 24 wks.	23.20	-3.40	-3.90	60,227	-13,537	-10,124
Psychiatric Disease *N* = 500, 15 wks.	76.90	-10.10	-7.60	38,450	-5,200	-3,800

**Table 4:** Impact of lean design workshops on changes in direct activity costs of the trial

Therapeutic Areas Number of Participants. Duration of Trials (wks.)	Baseline and Change in Direct Activity Cost					
	Per Participant			Total for Trial (,000)		
	Pre Workshop	Result of Workshop	Adopted	Pre Workshop	Result of Workshop	Adopted
Liver Disease *N* = 328, 78 wks.	$38,000	-$9,000	-$2,000	$12,333	-$2,821	-$394
Cardiac Disease *N* = 12,600, 250 wks.	$44,000	-$9,000	-$4,000	$556,920	-$119,700	-$57,960
Pulmonary Disease *N* = 164, 122 wks.	$345,000	-$28,000	$0	$56,516	-$4,595	$0
Cancer *N* = 1170, 128 wks.	$173,000	$0	$0	$202,176	$0	$0
Infectious Disease *N* = 2596, 24 wks.	$16,000	-$3,000	-$2,000	$41,796	-$7,179	-$6,231
Psychiatric Disease *N* = 500, 15 wks.	$30,000	-$4,000	-$1,000	$15,150	-$1,993	-$450

SoAs that were rebuilt from a simple ‘ground zero’ version generally retained fewer assessments. The types and reasons for simplification varied by protocol. Reconsideration of the value of some assessments led to their removal from the SoA. For examples, the realization that clinical assessments, such as physical examinations, did not generate data omitted them as data points in the SoA. Changes in the number and timing of some assessments were also made. For example, a complete blood count (CBC) done to screen for off-target effects of a drug that has early biological effects were recommended to be done at baseline and one or two early timepoints rather than every few weeks. In a few SoAs for large trials, a few assessments done to characterize the effects of the treatment were planned for smaller subsets of participants, generally done early in follow-up to inform whether there were any effects that needed continued collection.

The number and amounts of changes varied considerably across the trials (Tables 2, 3 and 4). Recommendations would have reduced participant hours by 4 to 47 per participant per trial.

The total changes in hours and costs for the trial were, as expected, proportional to the size and duration of the trial. For example, the $9,000 recommended reduction per participant in the cardiovascular trial with 12,600 participants followed for 250 weeks would save nearly $120 million for the whole trial. Few changes were recommended for the oncology trial. This reflected the highly standardized approaches to assess cancer progression in oncology trials.

After the workshops, study teams usually adopted some but not all the recommended revisions. This was expected because the workshops were considered exercises whose results were not mandatory. Adoption of changes was often limited because of the late stage of the SoAs that were reviewed. Even when there was no plausible biological reason that a treatment would influence a laboratory test or previous data showing no effect, some study teams were not comfortable with the uncertainty that some unexpected abnormal result might arise. Additionally, when asked, the quality of life and pharmacology groups simply asserted that no changes could be made in the number, frequency or sample size of PROs or PK-PD assessments (Table 5).

**Table 5:** Impact of lean design workshops on complexity of the trial

Therapeutic Areas Number of Participants. Duration of Trials (wks.)	Baseline and Change in Complexity (RVU)					
	Per Participant			Total for Trial		
	Pre Workshop	Result of Workshop	Adopted	Pre Workshop	Result of Workshop	Adopted
Liver Disease *N* = 328, 78 wks.	55.22	-14.93	0.00	55.22	-14.93	0.00
Cardiac Disease *N* = 12,600, 250 wks.	72.18	-12.49	-7.64	72.18	-12.49	-7.64
Pulmonary Disease *N* = 164, 122 wks.	444.79	-150.48	-41.71	444.79	-150.48	-41.71
Cancer *N* = 1170, 128 wks.	858.79	0.00	0.00	858.79	0.00	0.00
Infectious Disease *N* = 2596, 24 wks.	112.65	-18.38	-29.86	112.65	-18.38	-29.86
Psychiatric Disease *N* = 500, 15 wks.	77.74	-0.07	-1.12	77.74	-0.07	-1.12

Teams sometimes raised concerns about the potential importance of data for the FDA or other regulatory bodies. In general, they tried to anticipate FDA interests by including more assessments. When the agency did not comment on the assessments, the team assumed that the assessments had been approved and could not be changed. However, it was pointed out that ICH guidelines have recommended reductions in the amount of data collected in trials [1]. Additionally, teams may be better served by proposing a very lean version of the protocol and SoA and then adding elements back in if required by the agency. The items that were required by the FDA may reveal issues that have arisen in competitors’ trials.

## Discussion

There is a growing recognition that the increasing protocol complexity is unsustainable. The Lean Design workshops took the approach of working with individual study teams, starting over from a very basic Ground Zero SoA for collection of data. It challenged members of the study team to consider whether and when to include assessments and in how many participants, based on several principles of trial design and on the biology of the treatment. The process resulted in substantial potential savings in time, cost, and complexity of a set of actual protocols across several therapeutic areas. It also engaged the study team with the long term goal of encouraging them to think systematically and differently about the design of protocols and SoAs.

The workshops were described as exercises and that recommended changes were not mandatory. Protocols were generally in the late stages of development and approval, so many recommendations were not adopted. However, changes that were adopted would have resulted in substantial changes in the impacts of the revised SoA. For example, in the cardiovascular trial, adopting only $4,000 of the $9,000 recommended reductions in cost per participant would reduce the total cost of the trial by almost $58 million. Even when changes from the workshop were not adopted, team members often said that they agreed with the approach and the principles would inform their future development of SoAs.

More substantial changes identified in workshops would have been adopted if protocols had undergone the Lean Design exercise earlier in their development, ideally as the SoAs were being initially drafted. Feedback about the impact of design choices, provided by the Faro Smart Designer Tool may have the greatest impact when used in real time from the start of protocol design.

Importantly, the estimated costs and savings from the SoA pertained only to items listed in the SoA. They did not account for the fully loaded cost to the sponsor, including data collection, monitoring, cleaning, reconciliation, analysis, and reporting. Similarly, the ‘downstream’ impacts on third party and other sponsor activities were not assessed. This is an important consideration for panels of laboratory tests done for the purpose of assessing one or few of dozens of elements. The costs of monitoring and query resolution is also an important burden for clinic staff and sponsors. A comprehensive database of downstream costs of excess data points capture is in development.

There are various approaches to controlling the complexity of trials. Expecting review committees to make changes in the complexity of SoAs is not realistic because those reviews generally focus on major issues such as regulatory approval and competitive positioning. Some sponsors may try SoA templates, algorithms, or artificial intelligence to identify elements that are not essential. Artificial intelligence is being applied largely to recruitment of and selection, monitoring, and retention of participants [7–9]. There has been no description of its role in designing or improving the efficiency of SoAs. It is not yet clear whether AI could replace medical judgements about the value and patterns of assessments.

The face-to-face approach to rebuilding an SoA in this project may be more effective in making and retaining changes than just presentations about the principles. However, it is impractical for one committee or individual to review all trial protocols under development at a large pharmaceutical company. An approach of training several individuals in a company, including cross-functional leaders, such as biostatistics, to apply the methods, armed with a tool that provides feedback about the impacts of choices would facilitate broader effective adoption of Lean Design and its associated principles.

Simplification of trials across a company requires support and promotion from the leadership of the company and therapeutic areas. It requires buy-in from groups that can enable simplification including regulatory, clinical trial operations, and biostatistics. It is valuable to assess an effort to implement the system to quantify the impact of the efforts. This could reinforce the value of the implementation program. It requires estimating the change in the complexity and impacts of trials from trials designed before and after implementation of the program. It may also be valuable to occasionally reevaluate SoAs from trials to determine whether there might benefit from additional training.

Propelled by the results of the Lean Design workshops, Merck launched a company-wide project to implement its principles across all therapeutic areas. Simplification, including lean design principles, is considered in the initial design SoAs. Leaders from therapeutic areas are trained in the Lean Design principles to extend the effects to many trials in all therapeutic areas.

While the value of the Lean Design process has been demonstrated in one very large pharmaceutical company; the application and results may differ in other companies. The generalizability of the approach and results should be tested in other companies.

## Conclusion

We conclude that a process of rebuilding protocols from Ground Zero and according to a few principles and supported by quantitative feedback about the impacts of additions, may result in substantial reduction in the number of essential elements included in schedules of assessment with major reductions in the burdens and costs of clinical trials.
